# An audit of the dietary intake of Australian children with type 1 diabetes

**DOI:** 10.1038/s41387-018-0021-5

**Published:** 2018-03-09

**Authors:** Heather R. Gilbertson, Kristen Reed, Sarah Clark, Kate L. Francis, Fergus J. Cameron

**Affiliations:** 1 Department of Nutrition and Food Services, Royal Children’s Hospital, Melbourne, VIC Australia; 2 Department of Endocrinology and Diabetes, Royal Children’s Hospital, Melbourne, VIC Australia; 3 Clinical Epidemiology & Biostatistics Unit, Murdoch Children’s Research Institute, Melbourne, VIC 3052 Australia

## Abstract

To understand what children with type 1 diabetes in a representative tertiary hospital clinic are eating compared to their peers and explore dietary intake impact on HbA1c outcome. An open cross-sectional dietary audit of children and adolescents with diabetes aged 2–17 years attending the Royal Children’s Hospital, Melbourne was conducted using an age-appropriate validated Food Frequency Questionnaire. Total energy, macronutrient intake and diet quality were calculated and compared to dietary advice provided and national intake data. Body weight, and dietary intake influences on glycaemic control were investigated. Overall, 785 patients were recruited, from which 429 dietary surveys were completed. Dietary intakes were overall nutritionally adequate with macronutrient distribution (% total energy intake) being lower carbohydrate (48.6%), higher total sugars (22.4%), fat (32.9%), saturated fat (14.9%) and protein intake (19.1%) than recommendations, but similar to their peers. Energy intakes were excessive compared to their peers in the 4–13 year olds. Rates of overweight (30%) were significantly higher than national data (18%). Overall, 43% achieved optimal glycaemic control (HbA1c < 7.5%; <58 mmol/mol). HbA1c prediction via linear regression indicated that the following factors were associated with lower HbA1c values: being male, on pump regimen, lower rates of insulin per kg, shorter duration of disease. This audit has identified areas requiring targeted education/support to improve health outcomes including dietary adherence, rates of overweight/obesity, appropriate energy intakes and optimal glycaemic targets. Furthermore, it provides baseline data to evaluate efficacy of future interventions.

## Introduction

Nutritional management is one of the fundamental cornerstones of diabetes management to achieve optimal diabetes control, with HbA1c < 7.5% (<58 mmol/mol) associated with reduced risk of microvascular complications^1, 2^. Despite this emphasis on the importance of dietary education and management, the actual dietary intake of Australian children and adolescents with diabetes is not well documented.

Current dietary advice given to children and adolescents with diabetes is based on general healthy eating principles^3^. In Australia these principles are based on the Australian Dietary Guidelines for Children and Adolescents^4^ and is consistent with national^5^ and international^6^ guidelines. At diagnosis, children and their caregivers are provided with two formal diet education sessions. General healthy eating principles are taught with emphasis on eating regular meals and snacks based on low glycaemic index (GI), low fat, low sugar and high fibre food choices. Carbohydrate recommendations are based on ~50% of total energy intake, with an even distribution over the day, and particular attention given to quantity (carbohydrate counting) and quality (low GI food types).

Adherence to dietary advice is often reported as one of the most difficult aspects of diabetes management as impacts on the social and emotional aspects of eating. Previous studies report poor dietary compliance in American youths whose dietary intake substantially failed to meet national nutrition recommendations^7–9^.

National data for general population intake are available from the 2012 Australian National Nutrition Survey (NNS)^10^, which also combines data from the National Health Survey and the National Children’s Nutrition and Physical Activity Survey^11^ and are used to compare nutrient intake, physical activity levels and anthropometric measurements.

To date, limited evidence is available regarding compliance to dietary advice in Australian children with diabetes. A cross-sectional audit was undertaken in our clinic to determine the activity and dietary intake of our clinic patients. The information was compared to the dietary advice received and to age-matched National Nutrition Intake data to see how our children with diabetes were faring. With the dietary insight obtained from this audit, it is planned to identify areas of need for targeted nutritional management strategies/resources to improve health outcomes, minimise diabetes-related complications and improve the individual’s quality of life.

## Subjects and methods

An open, cross-sectional dietary audit of children and adolescents with diabetes who attended the Diabetes Clinic at the Royal Children’s Hospital Melbourne, Victoria was conducted over a six-month period between February–July 2014. Inclusion criteria included aged between 2–17 years inclusive, and diagnosed with diabetes. Exclusion criteria included patients <2 years or >18 years, and non-English speaking families who are unable to read/write English and therefore were unable to complete the FFQ.

Eligible participants were approached by the research dietitian while waiting for their appointment in clinic and invited to complete two questionnaires. Information statements were provided and written consent completed. All procedures in this study were approved by the Royal Children’s Hospital Human Research Ethics Committee (HREC 33214A).

The first questionnaire participants were asked to complete was the Australian Child and Adolescent Eating Survey Food Frequency Questionnaire (ACAES-FFQ). This is a self-administered, semi-quantitative 120-item questionnaire validated for ranking dietary intakes for children and adolescents aged 2–17 years^12^. Reproducibility and comparative validity analyses for this tool has been established^12, 13^. The ACAES-FFQ was completed by the child ≥12 years or alternatively by the parent/caregiver on behalf of the child. The ACAES-FFQ was sent to the University of Newcastle for scanning, and nutrient analysis was completed using FoodWorks (Version 3.02.581) to generate individual mean daily macro- and micronutrient intakes. Macronutrient intakes were compared to dietary advice provided and national intake data. Micronutrient intakes were compared to Recommended Daily Intakes^14^.

Data from the ACAES-FFQ were used to calculate an Australian Child and Adolescent Recommended Food Score (ACARFS) as a measure of overall diet quality, food variety and nutritional adequacy^15^, with a maximum potential score of 73. Scores ≥32 are considered good diet quality with reasonable diet variety, scores 19–31 indicate moderate diet quality, and scores ≤18 are indicative of poor diet quality with limited diet variety^15^.

The second questionnaire participants were requested to complete was an additional 17-item questionnaire that collected information on socioeconomic status, self-reported physical activity levels, engagement with diabetes healthcare team members, quality of carbohydrate intake and aspects of diabetes management including aspects of insulin adjustment, carbohydrate counting and frequency of self blood glucose monitoring.

A reply paid return envelope was provided to participants unable to complete the questionnaires in the given time, and followed up in their next clinic visit if the questionnaires had not been returned.

Baseline clinical data measures were collected from their recent diabetes visit. This included gender, age, diabetes type, duration of diabetes, relevant co-morbidities, insulin regimen and dosage, most recent HbA1c% (mmol/mol), height (cm) and weight (kg), from which *z*-scores for height, weight and BMI were subsequently calculated. All participant data were de-identified for inclusion in the study.

### Data analysis

Data are reported as mean ± SD for continuous variables and as proportions for categorical data. Mean BMI *z*-scores were calculated and categorised to allow a continuous version of the BMI score to be used in analysis. For the outcomes of BMI *z*-score and HbA1c value, univariate tests were conducted to determine whether there were differences in the sample based on sex, age and treatment regimen. Further, HbA1c value was predicted via linear regression. Data were analysed using Stata 14.2 (Stata/IC14.2, StataCorp 4905 Lakeway Drive, College Station, Texas 77845, USA).

## Results

### Participants

**Fig. 1:**
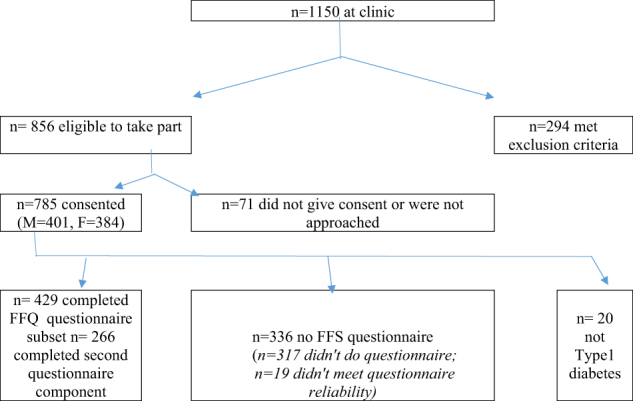
**Study consort diagram**

A total of 785 participants were consented into the study (Fig. 1). For the purpose of this report, only data for those with type 1 diabetes mellitus (*n* = 765) were included in this analysis. Three hundred and seventeen participants did not complete the ACAES-FFQ, and as per the guidelines for using the ACAES-FFQ^16^, participants reporting >18,000 kJ/day energy intake (*n* = 7) or with ≥5 incomplete responses (*n* = 12) were excluded. This resulted in a final sample of 429 children with type 1 diabetes reported for this paper (Table 1). Only 6/20 subjects with type 2 diabetes reliably completed the ACAES-FFQ which was considered too small a subset for comparison, hence their exclusion from this analysis.

**Table 1:** Demographic and clinical characteristics of the type 1 diabetes patients (*N* = 429)

	*N* (%)
Sex	
Male	217 (50.6%)
Female	212 (49.4%)
Age	
2–3 years	9 (2.1%)
4–8 years	92 (21.4%)
9–13 years	177 (41.3%)
≥14 years	151 (35.2%)
Treatment regime	
BD (twice daily)	157 (36.6%)
MDI (basal bolus)	139 (32.4%)
Pump (CSII^a^)	121 (28.2%)
Other (combination therapy)	12 (2.8%)
BMI *z*-score (*n* = 427)	
Mean (SD)	0.6 (0.9)
Hba1c value (*n* = 410)	
Mean (SD): full sample	7.9 (1.2)
2–3 years	7.7 (0.6)
4–8 years	7.7 (0.9)
9–-13 years	7.8 (1.0)
≥14 years	8.1 (1.5)
Duration of diabetes in months (*n* = 424)	
Mean (SD)	54.3 (40.6)
Insulin per kg (*n* = 419)	
Mean (SD)	1.0 (0.4)

^a^Continuous subcutaneous insulin infusion

When those who completed the ACAES-FFQ (*n* = 429) are compared to those who consented but did not complete the questionnaire (*n* = 317), it was found that non-completers reported having diabetes for a longer duration 62.5 months (95% CI: 57.8, 67.2) than completers (54.3 months; 95% CI: 50.4, 58.2, *p* = 0.008). Non-completers had higher insulin rate per kg of 1.05 (95% CI: 1.01, 1.09) compared to completers 1.00 (95% CI: 0.99, 1.05) (*p* = 0.033), higher HbA1c 8.10% (65 mmol/mol) (95% CI: 7.95, 8.24% or 63, 67 mmol/mol) compared with 7.88% (63 mmol/mol) (95% CI: 7.76, 8.00% or 61, 64 mmol/mol) (*p* = 0.020) and higher BMI *z*-score (0.79 (95% CI: 0.69, 0.89) versus 0.64 (95% CI: 0.56–0.73) (*p* = 0.021)).

### Dietary intake and quality

Mean energy and macronutrient intakes (carbohydrate, sugars, protein, total fat and saturated fat expressed as % of total energy) were calculated for gender and age groups for comparison to the National Nutrition Survey^17^ (Table 2). Energy intake increased with age from 2 to 13 years as expected. When comparing the current sample to the national sample there was evidence that the total nutrient intake (kJ) was greater in the study sample of 4 to 8 years olds who had a mean intake of 9933 kJ (95% CI: 9339, 10,526) compared with 7053 (*p* < 0.001); 9–13 year olds had a mean energy intake of 10159 kJ (95% CI: 9755, 10,563) compared with 8603 kJ (*p* < 0.001). Those aged 14 years or older reported overall lower energy intakes than the national sample 8986 kJ (95% CI: 8472, 9500) compared with 9158 kJ; however, there was no evidence that these values were substantially different (*p* = 0.509).

**Table 2:** Comparison of diet between Australian national data^10, 17^ and current study data

	*N*	Energy (kJ)	Protein (%)	Total fat (%)	Sat fat (%)	Total CHO (%)	Sugars (%)
Boys							
2–3 years							
T1 diabetes patients	5	5613.8	18.8	32.8	16.2	49	26.3
National data		6044.0	16.5	30.0	13.0	50.4	24.9
4–8 years							
T1 diabetes patients	51	9806.7	18.47	33.27	15.53	49.04	22.4
National data		7638.0	15.6	30.8	13.2	50.8	23.3
9–13 years							
T1 diabetes patients	81	10,655.5	18.67	32.79	15.11	49.17	22.3
National data		9209.0	16.2	31.3	13.0	49.9	21.7
≥14 years							
T1 diabetes patients	80	9929.0	19.94	33.61	15.38	47.16	21.4
National data		10,186.0	17.4	31.2	12.8	48.6	21.4
Girls							
2–3 years							
T1 diabetes patients	3	4026.7	18.67	39.67	18.67	43.33	21.7
National data		5850.0	16.7	30.4	13.8	49.9	24.1
4–8 years							
T1 diabetes patients	41	10,089.5	19.12	32.37	14.85	49.17	23.1
National data		6428.0	15.3	30.1	12.8	51.6	23.8
9–13 years							
T1 diabetes patients	96	9740.0	18.84	32.74	14.74	49.01	22.8
National data		7985.0	16.1	31.5	13.1	49.8	23.5
≥14 years							
T1 diabetes patients	70	7907.6	19.33	32.46	13.91	48.57	22.6
National data		8114.0	16.5	32.4	12.9	48.3	21.4
Total sample							
2–3 years							
T1 diabetes patients	8	5018.6	18.8	35.4	17.1	46.9	24.6
National data		5951.0	16.6	30.2	13.4	50.2	24.5
4–8 years							
T1 diabetes patients	92	9932.7	18.76	32.87	15.23	49.10	22.7
National data		7053.0	15.5	30.5	13.0	51.2	23.5
9–13 years							
T1 diabetes patients	177	10,159	18.76	32.76	14.91	49.08	22.6
National data		8604.0	16.1	31.4	13.1	49.8	22.6
≥14 years							
T1 diabetes patients	150	8985.7	19.65	33.07	14.69	47.82	22.0
National data		9158.0	17.0	31.8	12.8	48.5	21.4
All cases							
T1 diabetes patients		9601	19.07	32.94	14.94	48.60	22.4
National data		8059	15.8	31.0	13.5	50.3	22.5

Macronutrients reported as % of total energy intake

A high proportion of our children across the age groups met ≥80% RDI requirement for age (Table 3) for most micronutrients apart from children aged 2–3 years who were reported to have low intakes of iron and adolescents aged ≥14 years low in folate, calcium, magnesium and iron, comparable to the national data set^18^.

**Table 3:** Percentage of children with T1DM in the specified age groups meeting the RDI requirement (≥80% RDI) for micronutrient intake compared to the National Nutritional data^10, 18^

	T1D	NNS	T1D	NNS	T1D	NNS	T1D	NNS
Age group	2–3 years		4–8 years		9–13 years		≥14 years	
Thiamine	89	100	100	100	99	100	91	97
Riboflavin	89	100	100	100	99	100	95	99
Niacin equiv	67	100	100	100	100	100	97	100
Vitamin C	89	96	97	99	99	99	95	99
Folate	89	100	96	100	83	94	39	79
Vitamin A	78	100	99	99	97	94	70	83
Sodium	89	NR	100	NR	100	NR	99	NR
Potassium	78	NR	94	NR	94	NR	79	NR
Magnesium	89	100	99	100	98	94	63	55
Calcium	89	99	95	89	89	55	56	38
Phosphorus	89	100	100	100	94	88	91	93
Iron	33	99	91	100	98	99	68	94
Zinc	89	100	100	100	100	100	88	93

*NR* not recorded

Diet quality as measured by the Australian Dietary Guidelines was also considered. Our data indicated a better daily fruit intake compared to reported National data, but still suboptimal to recommendations. Overall, 78% of the 2–3 year olds reported eating the recommended 1 serve per day. This trend decreased with age with 68.5% of 4–8 years olds eating 1.5 serves per day (NNS 61% for this subgroup), 44.9% of 9–13 year olds eating 2 serves per day, and only 27.8% of >14 years olds eating 2 serves per day (versus only 1% reported in NNS for this age group).

The mean diet quality ACARFS score of the overall cohort was 30.6 ± 9.4, which is representative of moderate diet quality (Table 4). No differences were observed between males (30.5 ± 10.0) and females (30.6 ± 8.7; *t* = −0.06, *p* = 0.956). Diet quality scores were highest for 2–3 year olds 32.2 ± 7.9, similar for 4–8 year olds 31.7 ± 9.9, and 9–13 year olds 32.1 ± 9.2, whereas they were lower for those ≥14 years who scored 28.0 ± 8.9. Those aged 2–3 years differed from the other age groups with regard to: having a greater proportion of energy from core foods; and a lower proportion of energy from takeaway food. The individual subcategory ACARFS scores were also compared between each age group which included the ACARFS for intake of vegetables, fruit, meat, protein alternatives, grains, dairy, water and extras (Table 4). All age groups scored well below the total available score within each subcategory, with each score fairly consistent between the different age groups. The impact of insulin treatment modality on nutritional quality was also considered. Insulin treatment modality was recorded as either BD (twice daily injections of mixed insulin), MDI (multiple daily injections of four insulin injections per day using an insulin pen, also known as a basal bolus regime) or Pump (a continuous subcutaneous insulin infusion). In this sample those on BD had mean ACARFS score of 31 ± 10, MDI 30 ± 9, Pump 32 ± 8, indicating that those on insulin pump regimens demonstrated greater dietary variety and quality.

**Table 4:** Total and Food Group Australian Child and Adolescent Recommended Food Score (ACARFS) by age group in children with T1DM

ACARFS						Total
		Age groups				
	Total score available	2–3 years	4–8 years	9–13 years	≥14 years	
ACARFS vegetables	21	9.2 ± 5.1	9.3 ± 4.8	10.5 ± 4.6	9.2 ± 4.5	9.8 ± 4.6
ACARFS fruit	12	6.1 ± 3.3	6.2 ± 2.7	5.6 ± 2.8	4.4 ± 2.7	5.3 ± 2.8
ACARFS meat	7	1.8 ± 1.3	2.3 ± 1.2	2.4 ± 1.2	2.3 ± 1.3	2.3 ± 1.3
ACARFS alternate proteins	6	2.0 ± 1.1	1.5 ± 1.2	1.5 ± 1.2	1.5 ± 1.2	1.5 ± 1.2
ACARFS grains	13	6.0 ± 2.0	5.6 ± 1.8	5.5 ± 1.8	4.9 ± 1.8	5.3 ± 1.8
ACARFS dairy	11	5.6 ± 1.3	5.6 ± 2.2	5.1 ± 2.0	4.4 ± 1.9	5.0 ± 2.0
ACARFS water	1	0.6 ± 0.5	0.5 ± 0.5	0.7 ± 0.5	0.5 ± 0.5	0.6 ± 0.5
ACARFS extra	2	1.0 ± 0.9	1.3 ± 0.8	1.1 ± 0.8	1.0 ± 0.7	1.1 ± 0.8
ACARFS total	73	32.2 ± 7.9	31.7 ± 9.9	32.10 ± 9.2	28.0 ± 8.9	30.6 ± 9.4
Proportion of energy from core foods		75.9 ± 4.7	66.1 ± 10.6	64.7 ± 12.2	63.8 ± 11.9	64.9 ± 11.8
Proportion of energy from takeaway foods		4.3 ± 2.8	8.2 ± 4.0	8.4 ± 4.2	10.0 ± 5.3	8.8 ± 4.7

ARFS total scores ≥32 good diet quality; 19–31 moderate diet quality; ≤ 18 poor diet quality

There were 266 participant responses on the second questionnaire with regard to food product types, 58% reported selecting low to moderate glycaemic index (GI) bread types, 66% reported selecting low to moderate GI cereal types, 71% reported eating ordinary potato (not the low GI varieties) and 70% reported eating low GI Basmati rice for those who consumed these food types.

### BMI and body weight

Rates of overweight and obesity were also explored. BMI was calculated and analysed using BMI cut-off points from Cole^19^ to allow for direct comparison to the National data set^20^ (Table 5). Our survey reported higher proportion of participants in the overweight category and less in the normal category for both sexes across all age groups compared to the national data, and particularly the adolescent age group for boys 12–15 years and girls 12–17 years. When examining BMI *z*-score there was no evidence of difference between males and females (*p* = 0.073; 95% CI: 0.19, 0.14) or age groups (*p* = 0.676; 95% CI: −0.02, 0.01). There was weak evidence that the MDI (BMI *z*-score = 0.80) treatment regimen was associated with higher BMI score compared with BD (BMI *z*-score = 0.59; difference of 0.21 (95% CI: 0.004, 0.42)) and pump (BMI *z*-score = 0.51; difference of 0.29 (95% CI: 0.08, 0.49)).

**Table 5:** Comparison of BMI between study cohort and Australian national data^10, 20^

	*N*	Underweight (%)	Normal (%)	Overweight (%)	Obese (%)
Boys					
2–4 years					
T1 diabetes patients	5	0.0	60.0	20.0	20.0
National data		3.2	74.0	19.5	3.4
5–7 years					
T1 diabetes patients	32	3.1	62.5	31.3	3.1
National data		4.2	75.3	11.6	8.9
8–11 years					
T1 diabetes patients	68	2.9	60.3	26.5	10.3
National data		3.7	71.7	18.2	6.4
12–15 years					
T1 diabetes patients	75	5.3	61.3	55.3	8.0
National data		5.7	66.0	21.5	6.8
16–17 years					
T1 diabetes patients	35	5.7	65.7	20.0	8.6
National data		4.9	69.1	19.0	7.1
Girls					
2–4 years					
T1 diabetes patients	4	0.0	50.0	50.0	0.0
National data		5.8	71.5	15.9	6.8
5–7 years					
T1 diabetes patients	21	0.0	71.4	28.6	0.0
National data		5.9	66.3	19.7	8.2
8–11 years					
T1 diabetes patients	71	0.0	67.6	31.0	1.4
National data		4.8	67.1	21.1	7.0
12–15 years					
T1 diabetes patients	85	3.5	58.8	35.3	2.4
National data		6.4	68.8	17.4	7.4
16–17 years					
T1 diabetes patients	31	3.2	54.8	35.5	6.5
National data		7.4	68.8	15.5	8.2
Total sample					
2–4 years					
T1 diabetes patients	9	0.0	55.6	33.3	11.1
National data		4.4	72.8	17.8	5.0
5–7 years					
T1 diabetes patients	53	1.9	66.0	30.2	1.9
National data		5.0	70.9	15.5	8.5
8–11 years					
T1 diabetes patients	139	1.4	64.0	28.8	5.8
National data		4.2	69.5	19.6	6.7
12–15 years					
T1 diabetes patients	160	4.4	60.0	30.6	5.0
National data		6.1	67.4	19.5	7.1
16–17 years					
T1 diabetes patients	66	4.6	60.6	27.3	7.6
National data		6.0	69.0	17.4	7.6
All cases					
T1 diabetes patients	427	3.0	62.1	29.5	5.4
National data		5.1	69.8	18.2	6.9

### Physical activity level (PAL)

Self-reported PAL was collected in questionnaire two and was completed by 266 participants. With this consideration in mind, this subgroup reported an average of 82 min/day which meets the national recommendation of ≥60 min of physical activity every day^21, 22^. Overall, 62% of the cohort met this guideline as compared to 69% in the NNS. The mean time of physical activity was calculated for each participant using the sum of all self-reported physical activity (at school, outside of school and during leisure) divided by 7 for a daily total. There was no evidence that minutes of PAL was different between males (78.5 min/day) and females (85 min/day) (*p* = 0.305; 95% CI: 21.7, 6.84). Rates of reported activity were inversely related to age with 71% of 2–3 year olds, 61% of 4–13 year olds, and 62% of those ≥14 years meeting activity recommendations ≥60 min/day. There was no evidence that reported PAL (minutes per day) was associated with BMI *z*-score (*p* = 0.431; 95% CI: −4.9, 11.5) or age (*p* = 0.952 95% CI: −1.2, 1.1).

### Glycaemic control

Glycaemic control was evaluated using the most recent HbA1c measure. Mean HbA1c of this cohort was 7.9 ± 0.9% (63 ± 10 mmol/mol) which is consistent with the overall clinic average at that time. Cut-offs were used to determine the proportion of the cohort meeting best practice guidelines of an HbA1c ≤7.5% (<58 mmol/mol)^5, 6^. Categories included optimal control HbA1c ≤7.5% (<58 mmol/mol); moderate control >7.5% and ≤8.5% (>58 mmol/mol and ≤69 mmol/mol); and poor control HbA1c >8.5% (>69 mmol/mol). In our cohort, 43.4% had optimal glycaemic control, 31.5% moderate control and 25.1% poor control. Comparison of treatment regimen found that 54% of those on pump had optimal control, while 40% of those with BD and MDI had optimal control. There was evidence that being female compared to male was associated with higher HbA1c value (group difference of −0.25% (95% CI: −0.49, −0.02%), *t* = 2.1, *p* = 0.036). Increasing age was associated with 0.03% (95% CI: 0.01, 0.05) increase in HbA1c (*p* = 0.008).

### Predicting HBA1C

A series of univariate tests were run to determine which factors may account for variance in HbA1c including sex, age, insulin regimen, BMI *z*-score, insulin rate per kg, duration of diabetes, ACARFS score, percent of diet from protein/carbohydrate/fats/saturated fats and from ‘core foods’. (PAL was not included as only 60% of respondents completed questionnaire two.) Variables that were significant predictors after univariate testing were included in the model as independent variables (sex, age, insulin regimen, insulin rate per kg, duration of diabetes, ACARFS score, percent of dietary energy total fat intake). The model explained 15% of the variance in the data. When all variables are held equal the following variables are important predictors of HbA1c values: being female is associated with a 0.23% (95% CI: 0.02, 0.44) increase in HbA1c value; being on a pump regimen compared to a BD regimen is associated with a 0.33% (95% CI: 0.6, 0.01) reduction in HbA1c. For each unit increase in duration of diabetes (month), HbAc1 values increase by 0.007% (95% CI: 0.004, 0.010). For each unit increase in insulin rate per kg, there is a predicted increase of HbA1c by 0.62% (95% CI: 0.29, 0.95). Finally, the model indicates that for each percentage increase in dietary energy from total fat intake there is a 0.02% (95% CI: 0.00, 0.05) increase in HbA1c.

## Discussion

The dietary intake and associated aspects of diabetes management were examined in a large representative clinic population of children 2–17 years old with diabetes, and compared with National dietary recommendations and age-matched national nutritional intake data. Overall the dietary intake of this cohort was nutritionally adequate and met estimated energy requirements for growth and development, with the 4–13 year old subgroup reporting excessive energy intake and all age groups in the whole cohort having higher rates of overweight, but not obesity rates, compared to their peers. Overweight rates were particularly high in the adolescent age group in both sexes. Nutrition education needs to be targeted to address this phenomenon to avoid ‘overfeeding’ the diabetes to the health detriment of the individual. Dietary quality was observed to decline in both sexes >14 years with a mean ACARFS of 28. This follows similar trends reported in the literature regarding deterioration of diet quality and the rise of poor dietary habits with increasing age^23^.

Our children reported a greater intake of protein, fat, saturated fat, total sugars and energy compared to recommendations and their peers in the National Nutrition Survey^10^. Current recommendations for the distribution of macronutrient proportions are carbohydrate 50-55%, moderate sucrose intake (up to 10%), total fat 30%, saturated fat < 10% and protein 10–15% of total energy intake^6^. Our cohort consumed lower carbohydrate (48.6 ± 6.2%), higher total sugars (22.4 ± 5.6%), higher total fat (32.9 ± 4.8%), higher saturated fat (14.9 ± 3.1%) and higher protein intake (19.1 ± 3.3%) compared to recommendations. National Nutrition Data^10^ within the same age cohort reported carbohydrate (50.3%), total sugars (22.5%), total fat (31.0%), saturated fat (13.5%) and protein intake (15.8%). This suggests our children are eating higher protein/fat food sources at the expense of carbohydrate rich foods. Interestingly, the intake of simple sugars was almost the same between the current sample and the Australian sample, and this is consistent with other international findings^8, 9^. Overall diet quality and nutritional adequacy scored well in our clinic group, but declined with increasing age—also consistent with previous findings.

Participants were more overweight than their peers, with increased rates of overweight consistently being reported in other international diabetes centres.^24–28^. Although it is recognised that there is an increasing prevalence of overweight and obesity in the general population^29^, the increased reported rates of overweight in children with diabetes is concerning. Potentially too much emphasis is placed on daily glycaemic targets and increasing insulin doses to improve control and not enough focus on improving the quantity and quality of foods consumed. With increased awareness now of this association, dietetic interventions in the clinic setting can focus more specifically on this issue. Other clinicians in the multidisciplinary team may also be mindful of this inter-relationship before increasing insulin doses. The Diabetes Control and Complications Trial (DCCT) identified weight gain as an adverse consequence of intensive insulin therapy^1^. However, emerging evidence has shown improved glycaemic control can be achieved without inducing weight gain, through careful redistribution of insulin doses to more closely match requirements^30^. Children with diabetes can avoid substantial weight gain by optimising insulin management, adhering to individually tailored nutrition advice and regular participation in physical activities.

Only 43% of the cohort met the target for good glycaemic control which clearly needs to be addressed. The majority had optimal to moderate glycaemic control however this declined with the transition into adolescence, alongside diet quality. This places the adolescent population at increased risk of having nutritionally inadequate intakes, and has significant implications for the optimisation of their insulin therapy and overall diabetes treatment. Insulin pump therapy was associated with 0.33% improvement in HbA1c outcomes, however it should be noted that there may be bias in our clinic cohort as it tends to favour the highly motivated and better controlled individuals being considered for this treatment option.

The food frequency questionnaire is regarded as the most practical and economical dietary assessment method for the collection of comprehensive data in larger studies^31^. Issues for consideration when making comparisons with National Nutrition data include the different dietary methodology used for each survey (frequency versus 24-h recall and 3-day food records). Under-reporting is also likely in females ≥14 years as evidenced by our groups lower mean energy intake. Methodology was used to try and identify and exclude the over-reporters. Assessment of dietary intake presents unique challenges and current research suggests there is no single perfect method of dietary assessment as all methods are fraught with the potential of individual under- and over-reporting of intake^31, 32^.

This audit provides good baseline data for comparison of future targeted nutrition intervention and engagement strategies for our cohort to evaluate efficacy. A comprehensive, multidisciplinary approach to diabetes management is encouraged and this audit highlights the need to consider opportunities to review/expand service delivery models to improve engagement with team members and strive towards strategies to improve overall diabetes control. Dietitians must focus on weight management, specifically reducing dietary fat intake and improving the nutritional adequacy overall, with targeted intensive education to the 4–13 years old group to prevent excessive energy intake that is likely to be detrimental to diabetes-related health outcomes later in life. Additional education and support is required throughout adolescence to attempt to attenuate the decline in nutritional adequacy, diet quality, glycaemic control that is currently occurring with age, to minimise diabetes-related complications and improve quality of life outcomes. Dietary management for children of all ages with diabetes must always consider the medical management and be individually tailored to the highly variable and constantly changing nutritional needs of the growing child.
